# Physical Predictors of Favorable Postoperative Outcomes in Patients Undergoing Laminectomy or Laminotomy for Central Lumbar Spinal Stenosis: Secondary Analysis of a Randomized Controlled Trial

**DOI:** 10.3389/fneur.2022.848665

**Published:** 2022-04-15

**Authors:** Andrée-Anne Marchand, Mariève Houle, Julie O'Shaughnessy, Claude-Édouard Châtillon, Martin Descarreaux

**Affiliations:** ^1^Department of Chiropractic, Université du Québec à Trois-Rivières, Trois-Rivières, QC, Canada; ^2^Department of Anatomy, Université du Québec à Trois-Rivières, Trois-Rivières, QC, Canada; ^3^Department of Neurosurgery, Centre Intégré Universitaire de Santé et de Services Sociaux de la Mauricie-et-du-Centre-du-Québec, Trois-Rivières, QC, Canada; ^4^Division of Neurosurgery, Faculty of Medicine, University of Montreal, Trois-Rivières, QC, Canada; ^5^Department of Human Kinetics, Université du Québec à Trois-Rivières, Trois-Rivières, QC, Canada

**Keywords:** lumbar spinal stenosis, predictors, physical variables, postoperative outcome, prehabilitation

## Abstract

**Study Design:**

Secondary analysis of a randomized controlled trial.

**Objective:**

To identify preoperative physical variables associated with favorable postoperative outcome in individuals undergoing laminectomy or laminotomy for degenerative central lumbar spinal stenosis.

**Summary of Background Data:**

Clinical or condition specific variables have most commonly been studied as predictors of postoperative outcome in lumbar spinal stenosis. If associated to favorable postoperative outcome, modifiable physical variables would inform prehabilitation interventions for patients with degenerative central lumbar spinal stenosis.

**Methods:**

Patients awaiting surgery for central lumbar spinal stenosis were recruited to participate in a randomized controlled trial. Following baseline data collection of demographics, clinical portrait and physical testing, participants were randomized to either 6-week active prehabilitation program or hospital standard care. Complete baseline and postoperative data were obtained from 58 participants which were included in the present analysis. Favorable postoperative outcome was determined based on two outcome measures. Favorable outcome was defined as a decrease of ≥30% on the Numerical Rating Scale for leg pain intensity and a decrease of ≥30% on the Oswestry Disability Index for low back disability. Baseline physical variables were used to conduct binary logistic regression.

**Results:**

Sixty percent of participants were determined as having a favorable postoperative outcome. None of the included physical variables were found to be predictors of a favorable postoperative outcome based on leg pain intensity and low back pain-associated disability [trunk flexors muscle strength (OR = 0.73; 95%CI (0.02–27.12)] lumbar extensors muscle endurance [OR= 1.09; 95%CI (0.95–1.24)] total ambulation time [OR = 1.00 95%CI (0.99–1.01)] lumbar active range of motion in extension [OR = 1.08; 95%CI (0.95–1.23)] and knee extensors muscle strength [OR=1.02; 95%CI (0.98–1.06)].

**Conclusion:**

Results show that none of the investigated variables, all related to low back and lower limbs physical capacity, were predictors of postoperative recovery. Further testing in larger cohort is needed to assess the full potential of physical outcome measures as predictors of postoperative recovery.

## Introduction

Lumbar spinal stenosis (LSS) is the most common reason for undergoing lumbar spine surgery in adults over 65 years of age (1). While positive postoperative results support surgical interventions, with reports of patients experiencing rapid symptoms reduction, success rates over time are quite variable (2–8). In fact, one in three individuals will experience little to no benefit from surgery (9) and slightly more than 25% will require revision surgery within the 1-year (10). From a general standpoint, persistence of mild-to-moderate pain and disability up to 5-year should be expected (11, 12).

In the last decade, the perioperative teams have sought means to improve surgical outcomes and as a result, the concept of prehabilitation has emerged. Indeed, prehabilitation is defined as the process by which patients are better prepared to withstand the many stressors associated with surgery ahead of a surgical intervention. Augmentation of functional capacity and physiological reserve is the cornerstone of prehabilitation (13, 14). Therefore, the identification of individuals modifiable risk factors of poor surgical outcome and complications are necessary to tailor efficient prehabilitation interventions.

Recently, our group conducted a randomized controlled clinical trial investigating the effectiveness of exercise-based prehabilitation on improving postoperative recovery in patients awaiting surgery for central LSS compared to hospital standard care (15). While participants in the intervention group showed improvements in numerous clinical and physical outcome measures at the postintervention assessment, between group differences leveled out at the postoperative evaluation and follow-ups with the surgery having a tremendous positive effect on most patients in both groups. Considering that the main goal of prehabilitation is to facilitate recovery and return to baseline functional level, it was deemed important to identify physical predictors of favorable postoperative outcome that could in turn be targeted in future prehabilitation programs. Therefore, the objective of this study was to identify the physical variables associated with favorable postoperative outcomes following laminectomy or laminotomy surgery in patients with central LSS.

## Methods

### Study Design

This study is a secondary analysis of a randomized controlled trial investigating the effectiveness of exercise-based prehabilitation in patients awaiting surgery for lumbar spinal stenosis. A more detailed methodology regarding subject recruitment and data collection can be found in previous publications resulting from this trial (15, 16). The study protocol was registered (ClinicalTrials.gov: NCT02258672) and published elsewhere (17). Ethics approval for involvement of human participants was obtained through the Université du Québec à Trois-Rivières (UQTR) (CÉR-2014-008-00) and the Centre Intégré Universitaire de Santé et de Services Sociaux de la Mauricie-et-du-Centre-du-Québec (CER-14-204-07.07) institutional review boards. All participants provided written informed consent prior to data collection.

### Original Study

#### Participants

Background information on the original study is provided to establish the current study context. Sixty-eight participants were recruited from the Trois-Rivières regional hospital (Quebec, Canada) during outpatient clinical encounters with members of the neurosurgery team from February 2015 to June 2019. To be included, in the original study patients had to (1) be aged over 18 years, (2) have both clinical and imaging confirmation of degenerative primarily central lumbar spinal stenosis, (3) have opted to undergo decompressive surgery (open or minimally invasive approach), (4) be able to provide written informed consent voluntarily, and (5) to understand and speak fluent French. Potential participants were excluded if they presented with non-degenerative or other than primarily central canal stenosis, inflammatory arthritic conditions, vertebral instability requiring surgical non-instrumental or instrumental fusion, altered cognitive function, or any other conditions that made them unfit to participate in a rehabilitation program as judged by their treating neurosurgeon.

#### Intervention

**Figure 1:**
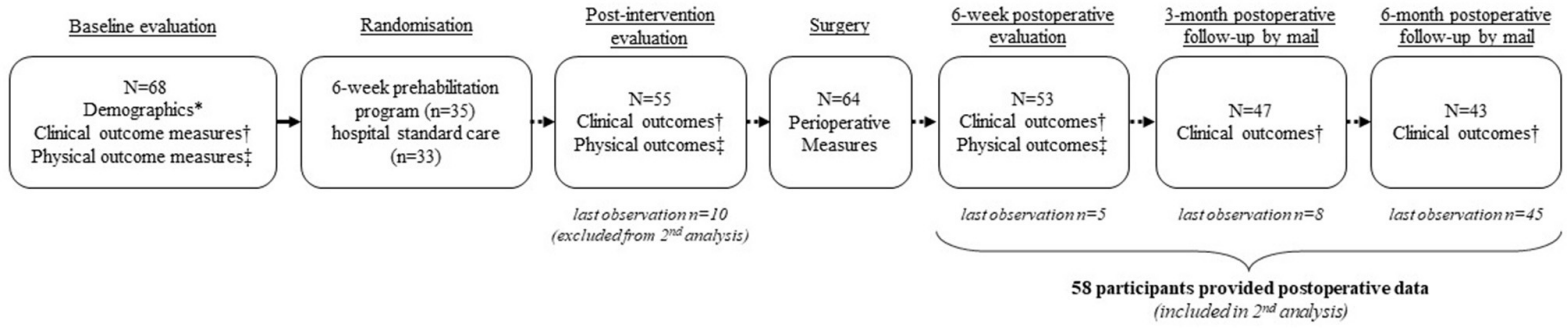
Timeline of outcome assessments and participants flowchart from the original trial. *Demographics included: Age, gender, employment status; variables included in the minimization process (smoking status; disability score ≥41%; presence of diabetes; nerve root motor disturbance confirmed by electrodiagnostic test); number of months since pain first started; presence of comorbidities; previous use of conservative care. †Clinical outcome measures included: Leg pain and low back pain intensity (Numerical Rating Scale); pain dominance (leg or back); low back pain-related disability (Oswestry Disability Index); Quality of life (European Quality of life EQ-5D); Kinesiophobia (Tampa scale of kinesiophobia); Depression (Beck Disability Index); Patients' global impression of change (at the post intervention assessment only). ‡Physical outcome measures included: Active lumbar ranges of motion; Lumbar flexor and extensor muscles maximum isometric voluntary contraction; Lumbar extensor muscles endurance (modified Sorenson test); Knee extensor muscles maximum isometric voluntary contraction; Walking capacities (time to first symptoms and total ambulation time during standardized treadmill evaluation).

In the original study, eligible participants were randomized to either a 6-week prehabilitation program or to hospital standard care. Upon enrollment, participants provided information on demographics, answered baseline self-reported questionnaires and completed physical tests. A detailed description of outcome measures can be found elsewhere (17). All evaluation sessions were conducted at the UQTR biomechanics laboratory and followed a standardized format. Figure 1 presents the timeline of assessments along with data collection for every evaluation time point. Participants randomized to the hospital standard care group did not receive any particular intervention nor were they discouraged from keeping up with current physical activities if any. Participants randomized to the prehabilitation group aimed to meet one-on-one with the kinesiologist three times a week for 6 weeks. Training sessions lasted 30 mins and included a 5-mins warm-up and a set of five exercises designed to improve trunk stabilization, posterior chain muscles strength and endurance, and lower limb and hip muscles strength. A detailed progression of the proposed exercise can be found elsewhere (16). The analysis of the intervention effectiveness on clinical outcomes and physical function showed that despite statistically and clinically significant changes in favor of the prehabilitation group seen at the preoperative assessment these differences were not maintained at the postoperative and follow-up assessments (15).

### Current Study

In the present study, we aimed to identify preoperative physical variables that are associated with favorable postoperative outcomes in a sample of patients who underwent surgery for degenerative central lumbar spinal stenosis. Of the 68 participants originally enrolled in the main trial, 58 provided both baseline and postoperative data and were included in the secondary analysis. Of the 10 participants excluded from this secondary analysis, four opted out of surgery and six did not provide postoperative data. Their baseline characteristics were, however, similar to those of completers. Postoperative outcomes were dichotomized into “favorable” or “unfavorable” based on whether participants reached a clinically meaningful percent change score from baseline in two independent outcome measures (18, 19). Favorable outcome was defined as a decrease of ≥30% from baseline in both leg pain intensity on the Numerical Rating Scale (NRS) and in low back pain-associated disability on the Oswestry Disability Index (ODI) (18, 19). Considering that participants' response rate varied over time, the latest timepoint at which they each provided data was used to determine the postoperative outcome (see Figure 1 for the participants flowchart). Thus, five participants last provided data at the 6-week postoperative evaluation, eight last provided data at the 3-month postoperative follow-up and 45 participants last provided data on the 6-month postoperative follow-up. The 13 participants that did not complete all postoperative follow-ups differed from the 45 completers in that they had lower active range of motion in lumbar extension (−6.5°; *p* = 0.009) at baseline. This finding is, however, unlikely to explain the loss of these participants to follow-up.

### Outcome Measurement

Pain intensity was assessed using an 11-point NRS. Each patient subjectively rated its current level of leg pain on a scale of 0 to 10 (0 being an absence of pain 10 being the worst pain imaginable) (20).

Low back-related disability was measured using the validated French version of the ODI questionnaire (version 2.1a) (21). The ODI comprises 10 questions related to daily activities, including pain intensity, personal care, lifting, walking, sitting, standing, sleeping, sexual life, social life, and traveling. Each question is rated on a scale of zero to five points with a maximum possible score of 50 which in turn is reported as a percentage. Higher scores indicate greater disability.

### Physical Variables Measurement

Active lumbar ranges of motion were assessed with a digital inclinometer (Digital Dualer IQ Pro™, Model CM101; JTECH Medical, Midvale, UT, USA) (22). Each range of motion (flexion, extension, right lateral flexion, left lateral flexion) were measured twice and averaged for the analysis.

Lumbar extensor muscles endurance was assessed with a modified version of the Sorenson test using an inclined bench (23). Participants were positioned on a 30° inclined Roman chair, the iliac crest aligned with the chair's border, the upper body maintained in a horizontal position (parallel to the floor) and the arms crossed over the chest. The position was maintained for as long as possible and the test was stopped when participants were either no longer able to maintain a proper horizontal position (as externally judged by the assessor), became too fatigued to continue, or experienced pain. Fatigue was objectively measured using a perceived level of effort using a Borg's Scale (24).

Isometric strength of the knee extensor muscles was assessed using a load cell (Model LSB350; Futek Advanced Sensor Technology Inc., Irvine, CA, USA) (25). Participants sat on a bench with both hips and knees bent at 90°. Three trials were completed for each leg. The highest result of each leg was recorded and averaged for analysis.

Strength of lumbar flexor and extensor muscles was assessed using an isokinetic testing device (LIDO, Loredan Biomedical Inc., Davis, CA, USA) (26). Each measurement was taken three times and averaged for analysis.

Walking capacities were assessed using two components of the exercise treadmill examination: the time to first symptoms (TFS) and the total ambulation time (TAT). Both variables were measured at a walking speed of 1.2 mph, on a 4° inclined ramp. The examination was stopped at the onset of severe symptoms, defined as the level of discomfort that would cause the patient to stop walking in usual life situations.

### Data Analysis

Given that in the original study, both groups had similar postoperative improvements, all eligible participants (*N* = 58) were included regardless of initial group allocation for the present analysis. As the main goal was to identify a set of predictive variables, we did not use imputed data for this study. Each baseline variable was assessed for normality of its distribution using the Shapiro-Wilk test and visual inspection of the corresponding histogram. Means and standard deviations were calculated for continuous variables while proportions were reported for dichotomous variables for the complete sample of participants. Multivariate binary logistic regression was conducted to identify potential predictors of postoperative favorable outcome using baseline physical variables. Because of the small number of cases available for the conduct of the regression analysis, we determined a set of five candidate variables based on their potential to be modified by exercise-based prehabilitation interventions. In addition, leg pain dominance was entered into the regression model, despite not being a physical variable, given that it is known to be the strongest predictor of postoperative recovery. We tested for collinearity with variance inflation factor (VIF) and considered value ≥ 5.0 as indicative of collinearity (27). We controlled for randomization group, leg pain intensity and low back-related disability. Adjustments for overfitting and evaluation of the model were performed by bootstrap. Nagelkerke *R*^2^ was used to describe the amount of variation explained by the independent variables in the model. Model results were reported with odds ratios (OR) and 95% confidence intervals (95% CI). All significance tests were two-sided and conducted at a 5% significance level. All statistical analyses were performed using IBM SPSS Statistics version 28 (Armonk, NY: IBM Corp.).

## Results

Baseline characteristics for all participants are presented in Table 1. Sixty percent (*n* = 35) of the sample achieved a favorable outcome as determined by the model prerequisites.

**Table 1:** Participants' baseline characteristics.

	**All participants** **(*N* = 58)**
**Demographics**	
Age—years (mean; SD)	69.1 (7.9)
Gender, female—*n*(%)	24 (41.4)
BMI—kg/m^2^ (mean; SD)	28.7 (4.8)
*Minimization criteria*	
Smoker—*n* (%)	24 (41.4)
Diabetes—*n* (%)	9 (15.5)
ODI≥41%—*n* (%)	20 (34.5)
Positive EMG findings—*n*(%)	8 (15.1)
**Clinical variables**	
Pain dominance	
Leg—*n* (%)	46 (79.3)
Back—*n* (%)	12 (20.7)
Leg pain intensity—/10 (mean; SD)	7.1 (2.2)
Back pain intensity—/10 (mean; SD)	5.4 (2.9)
Back disability—/100 (mean; SD)	36.8 (14.7)
Kinesiophobia—/68 (mean; SD)	46.2 (8.2)
Depression—/63 (mean; SD)	4.4 (4.0)
Quality of life—(item 1, 2, 3) *n* (%)	
EQ-5D mobility	0(0); 9(15.5); 49(84.5)
EQ-5D autonomy	0(0); 45 (77.6); 13(22.4)
EQ-5D activity	11(19); 41(70.7); 6(10.3)
EQ-5D pain	1(1.7); 41(70.7); 16(27.6)
EQ-5D anxiety	0(0); 33(56.9); 25(43.1)
**Physical variables** (mean; SD)	
Active lumbar ROMs—degrees	
Flexion	67.6 (22.9)
Extension	14.5 (6.4)
Right lateral flexion	13.8 (7.6)
Left lateral flexion	12.6 (6.8)
Trunk muscles strength—*N* m	
Flexion	45.6 (25.8)
Extension	29.3 (28.2)
Knees extensors strength—lbs	61.7 (30.7)
Lumbar extensors endurance—s	42.5 (55.2)
Walking capacities—s	
Time to 1st symptoms	109.9 (91.5)
Total ambulation time	191.4 (120.5)

*BMI, Body mass index; ODI, Oswestry Disability Index; EMG, Electromyography; ROM, Range of motion*.

We identified from previous effectiveness analyses (15) the baseline physical variables that were modified by the prehabilitation intervention (i.e., for which between-group significant change was found at the postintervention assessment). These variables were (1) trunk flexors muscle strength; (2) lumbar extensors muscle endurance; (3) total ambulation time (4) lumbar active range of motion in extension; and (5) knee extensors muscle strength. These five physical variables were considered candidate prognostic factors and entered into the regression model along with leg pain dominance. There was no collinearity found between the included variables with VIF values ranging from 1.05 to 2.71. The prediction model retained leg pain dominance as the only significant independent predictor of postoperative favorable outcome. The independent variables explained 29.0% of the model variance. Table 2 presents the multivariate binary logistic regression results.

**Table 2:** Result of the multivariate binary logistic regression analysis.

**Variable**	**Odds Ratio (95% CI)**	***p***	**Multivariate regression**
Trunk flexor muscles strength	0.73 (0.02–27.12)	0.80	*R*^2^ = 0.29
Lumbar extensor muscles endurance	1.09 (0.95–1.24)	0.80	
Total ambulation time	1.00 (0.99–1.01)	0.43	
Active ROM in lumbar extension	1.08 (0.95–1.23)	0.21	
Knees extensors strength	1.02 (0.98–1.06)	0.19	
Leg pain dominance	**1.36 (1.03–1.78)**	**0.02**	

*CI, Confidence interval; ROM, Range of motion; bold denotes statistical significance*.

## Discussion

The aim of this study was to identify physical variables associated with favorable postoperative outcome in individuals undergoing decompressive surgery for central lumbar spinal stenosis. This is one of the few studies to investigate whether postoperative favorable outcome based on clinically meaningful difference can be predicted by objectively measured physical variables. Surprisingly, none of the considered physical variables were found to be associated with a favorable postoperative outcome based on leg pain intensity and low back pain-associated disability.

Numerous prognostic factors have been studied in the context of LSS surgery in relation to just as many outcomes. A systematic review of preoperative predictors for LSS surgery have investigated 21 prospective studies and reported on the predictive value of different outcome measures (28). Although there was a clear variation in the number of predictors and outcome measures used, the authors concluded that at 6-month follow-up, preoperative expectations predicted subjective outcome and being a male and of younger age predicted better postoperative walking ability. Furthermore, at 2 to 5-years follow-up, better preoperative walking capacity predicted better postoperative capacity and satisfaction. On the other hand, preoperative depression predicted higher levels of pain, less treatment satisfaction, poorer walking capacity and less global satisfaction (28).

More recently, additional studies have reported on numerous categories of predictors of good but also poor surgical outcomes in LSS. For instance, radiological severity of the stenosis at the laminectomy level was not predictive of surgical outcome at 1 to 5-year after instrumented posterior decompression (29) and at 1-year after surgically implanting interspinous device (30). Conversely, severe central stenosis and single-level central stenosis have been associated with lower pain intensity and higher satisfaction at 2-year follow-up (31).

With regards to clinical outcomes, higher preoperative disability has been described as a predictor of better outcome at 2-year follow-up whereas a history of psychiatric disease have been associated with a worse disability outcome (32). Similarly, depressive symptoms were strong predictors of poorer disability, symptom severity, walking capacity, and health related quality of life outcomes at 1-year follow-up (33, 34). In addition, symptoms duration of more than 33 month has been associated to a less favorable functional outcome at one- and 2-year follow-ups (35) whereas patients with symptoms of fewer than 12-month duration experience significantly better outcomes at 4-year follow-up (36).

With respect to objective physical outcome measures, Lee et al. reported that radiculopathy confirmed by electrodiagnostic study was related to unsuccessful surgical outcomes (37). In addition, Shen et al. reported on the predictive value of hand grip strength and found that higher preoperative values were associated with better surgical outcomes in terms of disability and health status 6-month after spine surgery (38).

To the best of our knowledge very few studies have investigated objective and modifiable physical measures related to the low back or lower limbs to predict surgical outcome. Although self-reported measures may be more easily collected in the clinical setting, objective physical measures offer valuable insight on patients' current level of physical fitness and facilitate the identification of deficits that could then be targeted in prehabilitation programs. Among modifiable physical predictors of postoperative success, isometric trunk extensor strength was reported to be associated with 6-month postoperative 6-mins walk distance in patients undergoing surgery for LSS (12). Furthermore, gait measurements derived from a smart-shoe technology showed promising results for predicting postoperative pain intensity and low back disability outcomes (39). Finally, a preoperative body mass index ≤ 29.1 kg/m^2^ was reported to be associated with higher surgical success at 2-year follow-up (40) and the presence of skeletal low muscle mass has been shown to be a significant predictor of falls within 12-months of surgery (41).

Variability in predictive value across studies may be attributable to the wide definitions used to define “favorable outcome,” “success” or “satisfaction” following surgery, the inclusion of mixed surgical populations such as the use of complex surgical techniques, the differences in the choice of outcome measures and whether the latter were specific to the target population, and self-reported or objectively measured. The fact that numerous predictors have been identified based on different outcomes suggests that their predictive value may be outcome specific. Therefore, there is a need for a consensus about a core set of relevant outcomes to measure postoperative success in LSS which in turn would increase comparability across studies. Larger studies are needed to assess the full potential of physical variables as predictors of postoperative favorable outcome in patients with lumbar spinal stenosis and to inform the design of more effective prehabilitation programs.

### Limitations

Due to losses to follow-up, timing of outcomes measurement differed between participants, and it was therefore not possible to consider the postoperative trajectory as a whole to determine outcome. Determining predictors of outcome based on the combination of two different intervention groups may be viewed as a limitation, but the fact that they both yielded similar postoperative outcomes made the grouping possible. The small sample size resulted in great variability for many predictive variables limiting the power to find significant associations with the postoperative outcome and only a small number of potential predictive variables could be tested. Similarly, the inclusion of participants with low back pain dominance, considered non-optimal surgical candidate as opposed to leg pain dominance, may have affected our ability to identify predictive factors of favorable postoperative outcome.

## Conclusion

This study examined physical variables that may influence postoperative outcome in individuals with lumbar spinal stenosis. Results show that none of the investigated variables, all related to low back and lower limbs physical capacity, were predictors of postoperative recovery. Further investigation of modifiable physical variables able to predict surgical outcome in LSS is needed to define efficient prehabilitation interventions for this population.

## Data Availability Statement

The raw data supporting the conclusions of this article will be made available by the authors, without undue reservation.

## Ethics Statement

The studies involving human participants were reviewed and approved by the Research Ethics Board of Université du Québec à Trois-Rivières (CÉR-2014-008-00) and the Centre Intégré Universitaire de Santé et de Services Sociaux de la Mauricie-et-du-Centre-du-Québec (CER-14-204-07.07). The patients/participants provided their written informed consent to participate in this study.

## Author Contributions

AAM took part in the conceptualization and the design of methodology, conducted the investigation and analyses, and wrote the initial draft. MH was involved in conducting the investigation and reviewing and editing the manuscript. JO and CÉC took part in the conceptualization of the study, provided resources during the data collection, and reviewed the manuscript. MD was responsible for project administration and edited and reviewed the manuscript. All authors contributed to the article and approved the submitted version.

## Funding

This study was provided by the Chaire de Recherche Internationale en Santé Neuromusculosquelettique and its partner and the Centre Intégré Universitaire de Santé et de Services Sociaux de la Mauricie-et-du-Centre-du-Québec. The contribution of AAM was supported by the Fonds de recherche du Québec en Santé (FRQS).

## Conflict of Interest

The authors declare that the research was conducted in the absence of any commercial or financial relationships that could be construed as a potential conflict of interest.

## Publisher's Note

All claims expressed in this article are solely those of the authors and do not necessarily represent those of their affiliated organizations, or those of the publisher, the editors and the reviewers. Any product that may be evaluated in this article, or claim that may be made by its manufacturer, is not guaranteed or endorsed by the publisher.
